# The Emerging Role of Mast Cells in Response to Fungal Infection

**DOI:** 10.3389/fimmu.2021.688659

**Published:** 2021-06-03

**Authors:** Miao Yu, Xiao-ting Song, Bo Liu, Ting-ting Luan, Shuang-lu Liao, Zuo-tao Zhao

**Affiliations:** ^1^ Department of Dermatology and Venerology, Peking University First Hospital, Beijing, China; ^2^ Beijing Key Laboratory of Molecular Diagnosis on Dermatoses, Beijing, China; ^3^ National Clinical Research Center for Skin and Immune Diseases, Beijing, China; ^4^ Peking University School of Nursing, Beijing, China

**Keywords:** mast cell, fungi, pathogen-associated molecular patterns, *Candida albicans*, *Aspergillus fumigatus*, *Sporothrix schenckii*

## Abstract

Mast cells (MCs) have been considered as the core effector cells of allergic diseases. However, there are evidence suggesting that MCs are involved in the mechanisms of fungal infection. MCs are mostly located in the border between host and environment and thus may have easy contact with the external environmental pathogens. These cells express receptors which can recognize pathogen-associated molecular patterns such as Toll-like receptors (TLR2/4) and C-type Lectins receptors (Dectin-1/2). Currently, more and more data indicate that MCs can be interacted with some fungi (*Candida albicans*, *Aspergillus fumigatus* and *Sporothrix schenckii*). It is demonstrated that MCs can enhance immunity through triggered degranulation, secretion of cytokines and chemokines, neutrophil recruitment, or provision of extracellular DNA traps in response to the stimulation by fungi. In contrast, the involvement of MCs in some immune responses may lead to more severe symptoms, such as intestinal barrier function loss, development of allergic bronchial pulmonary aspergillosis and increased area of inflammatory in *S. schenckii* infection. This suggests that MCs and their relevant signaling pathways are potential treatment regimens to prevent the clinically unwanted consequences. However, it is not yet possible to make definitive statements about the role of MCs during fungal infection and/or pathomechanisms of fungal diseases. In our article, we aim to review the function of MCs in fungal infections from molecular mechanism to signaling pathways, and illustrate the role of MCs in some common host-fungi interactions.

## Introduction

Fungal infections are a serious health problem all over the world. Currently, more than 300 million people suffer from severe fungal infections, and an estimated over 1.5 million people die from deep fungal infections each year (1). With broad-spectrum antibiotics used and the increase of immunodeficiency disease such as acquired immune deficiency syndrome, pathogenic fungi opportunistic infections showed a trend of rising sharply (2–4). Although early diagnosis and the use of antifungal drugs have a certain positive effect on the prevention and control of fungal infection, there are still some unsolved problems in clinical practice, such as insufficient evidence of some etiology and drug resistance.

Both innate immunity and adaptive immunity play an indispensable role in anti-fungal infection. Mast cells (MCs) are one of the most important innate immune cells, which can initiate and regulate innate and adaptive immunity. Traditionally, MCs have been referred as the key effector cells of allergic diseases, such as urticaria, allergic rhinitis and bronchial asthma (5–7). MCs are mostly located in the border between host and environment such as skin, gastrointestinal and respiratory mucosa and thus may have easy contact with the external environmental pathogens (8). In addition, MCs express a variety of different pattern recognition receptors (PRRs) on their cell surface to detect bacterial, viral, fungal or parasitic components known as pathogen-associated molecular patterns (PAMPs) (9) such as β-glucan, mannan and lipopolysaccharide. Due to their strategic location and the wide variety of PRRs they express, many researchers have attempted to explore the potential immune relationship between MCs and fungal infection (9–11). Therefore, we will review the role of MCs in fungal infections from molecular mechanism to signaling pathways, and illustrate the role of MCs in some common fungal infections (e.g., *Candida albicans* (*C. albicans*), *Aspergillus fumigatus* (*A. fumigatus*) and *Sporothrix schenckii* (*S. schenckii*)) to light new ideas for prevention and treatment.

## Fungi Recognition Receptors on MCs

MCs can directly recognize PAMPs *via* their PRRs and then be activated. PRRs have comprised mainly five families including Toll-like receptors (TLRs), C-type lectin-like receptors (CLRs), Nucleotide-binding oligomerization domain (NOD)-like receptors (NLRs) and retinoic acid-inducible gene I (RIG-I) like receptors (RLRs) and absent-in-melanoma (AIM)-like receptors (ALRs) (12). Current studies have shown that TLRs and CLRs are main PRRs in host anti-fungal infection. The fungal wall is composed of many layers. Any component of the cell wall could be a potential PAMP. It is reported that the main molecules that trigger the immune response are chitin, β-glucan, mannan and others (13).

### Toll-Like Receptors (TLRs)

The TLR family is an important group of receptors that recognize invading microorganisms through innate immunity. A total of 13 members of the TLRs family have been reported, 10 of which are associated with human including TLR1-10 (14). TLRs are distributed on a variety of cells including macrophages, dendritic cells and MCs. Mouse connective tissue-type MCs (CTMC) express TLR2, TLR4, and TLR5 on cell surface membrane, whereas TLR3, TLR7, and TLR9 are present both on the cell membrane and intracellularly (15). TLRs can recognize and be activated by different PAMPs. Compared with bone marrow-derived MCs (BMMCs) from wild type mice C57BL/6, a decrease in *C. albicans* phagocytosis and nitric oxide (NO) production were detected in TLR2^-/-^ mice (16). Whereas mannan, extracted from *Saccharomyces cerevisiae*, directly activates murine CTMC to initiate the proinflammatory response *via* TLR4 (17). When pretreated with TAK242, a small-molecule-specific inhibitor of TLR4 signaling, CTMC significantly decreased the generation of cysLT and reactive oxygen species (ROS) as compared to nontreated MCs in response to mannan (17). In summary, TLR2 and TLR4 are the main members of TLRs on MCs with potential to bind different PAMPs on the surface of fungi and induce subsequent host immune response.

### C-Type Lectin-Like Receptors (CLRs)

CLRs are a family of calcium dependent receptors that bind to carbohydrates. A total of 17 members have been reported. Lectin activity of these receptors depends on conserved carbohydrate recognition domains. CLRs include Dectin-1, Dectin-2, macrophage-inducible C-type lectin (Mincle), intercellular adhesion molecule (ICAM-3) and dendritic cell-specific ICAM3-grabbing non-integrin (DC-SIGN) (18). Studies on CLR expression and immune function in MCs are still limited. Dectin-1 is the most commonly studied receptor in CLRs, which exists in both human and murine MCs. Dectin-1 can recognize β-glucans and trigger a variety of cellular responses such as phagocytosis and the production of cytokines. Zymosan, a β-glucan derived from yeast of *Saccharomyces cerevisiae*, for example, induced mature mice peritoneal MCs migration and pro-inflammatory expression *via* Dectin-1 (19). Similar results were observed in human cord blood-derived mast cells (CBMCs). When incubated with zymosan, CBMCs significantly decreased the level of leukotriene (LT) B_4_ and C_4_ in the presence of inhibitors of Dectin-1 (20). Some studies have shown that Dectin-2 can activate FcRγ chain to recognize α-mannose and induce immune response in dendritic cells (21), macrophages (22), and neutrophils (23), which leads to upregulation of TNF-α and IL-1ra secretion (24). Host Dectin-2 preferentially binds to hyphae form of *C. albicans, Microsporum audouinii* and *Trichophyton rubrum (* 24), which is essential for inducing Th1/Th17 immune responses (25). The expression and immune function of Dectin-2 of MCs remains to be further studied.

## Signaling Pathways of MCs Against Fungi

### TLR Signaling Pathway

TLR signaling pathway can be mainly divided into two different mechanisms, namely Myeloid differentiation 88 (MyD88) -dependent pathway and MyD88-independent pathway. The MyD88 dependent pathway is used by all TLRs except TLR3 (26). MyD88^-/-^ mice showed an increase of susceptibility to *C. albicans* infection and the production of proinflammatory cytokines such as TNF-α, IFN-γ and IL-12p70 could not be detected from antigen-stimulated splenocytes (27). Upon activation, MyD88 might trigger several different intracellular signaling pathways such as nuclear factor kappa-B (NF-κB), mitogen activated protein kinases (MAPK including extracellular signal-regulated kinase (ERK), p38 and c-Jun N-terminal kinase (JNK)) and phosphatidylinositol-3 kinase (PI3K), leading to the release of inflammatory cytokines and other inflammatory mediators. IgE-sensitized BMMCs from both TLR2 and MyD88 gene-deleted mice showed a decrease of IL-6 production in comparison of wild type BMMCs (28). Furthermore, a high concentration of *Malassezia sympodialis* extract inhibited phosphorylation of ERK1/2 and the release of IL-6 in IgE-sensitized BMMCs, indicating that the ERK1/2 pathway may regulate release of IL-6 (28). Except for ERK1/2, other molecules which involved MyD88-dependent pathways for TLR activation have been less investigated in MCs against fungi. PI3K pathway has been reported to be involved in lipopolysaccharide stimulation of murine BMMCs to produce IL-6 and TNF-α (29). In other cells, such as oral epithelial cells, all *Candida* species were able to activate the NF-κB and c-Jun pathway and only *Candida dubliniensis* and *C. albicans* hyphae can trigger MAPK/MKP1/c-Fos pathway (30).

### CLR Signaling Pathway

CLR signaling pathway can be divided into tyrosine kinase Syk-dependent and -independent signaling pathways. Both signaling pathways eventually activated NF-κB, and produced cytokines and chemokines such as IL-6, IL-23, TNF-α, macrophage inflammatory protein and CXCL12, which affected differentiation of Th1 and Th17 cells. Traditionally, CLRs (e.g., Dectin-1, Dectin-2) are coupled with Syk to further activate NF-κB pathway by activating caspase recruitment domain protein 9 (CARD9) in Syk-dependent signaling pathway. CARD9 was expressed on the surface of MCs, but its expression level was slightly lower than that of macrophages, dendritic cells and neutrophils. It was found that LTC4 and LTB4 were released from human MCs co-cultured with zymosan, suggesting that the expression of LTC4 and LTB4 was mediated by Dectin-1/Syk pathway (20). In Syk-independent signaling pathway, activated CLRs (e.g., Dectin-1 and DC-SIGN) can recruit GTPase Ras proteins which subsequently activate serine/threonine-protein kinase Raf-1. This results in Raf-1 mediated phosphorylation of the p65 subunit of NF-кB, which leads to the production of cytokines (31). In addition, the interaction between PRR can be more effective in the control of fungal infection. Studies have been proved that Dectin-1 and TLR2 collaborate to enhance the role of MAPK pathway resulting in an increase of cytokines (32).

## MCs and Fungi

### MCs and *C. albicans* Infection

*C. albicans* is one of the most common dimorphic fungus colonizing mucosal surfaces such as gastrointestinal tract,oral-nasal cavity and skin. Vulvovaginal candidiasis is one of the most common forms of candidiasis. When host immunity is suppressed or damaged, *C. albicans* can result in severe invasive diseases. Invasive candidiasis is the most common critical care-associated fungal infection in patients hospitalized in intensive care units, with mortality rates between 40% and 55% (33).

MCs play a positive role in the defense mechanism against *C. albicans* infections, including phagocytosis and killing yeasts in the extracellular environment (34). Both yeast and hyphae of *C. albicans* can induce the degranulation of BMMCs and lead to the production of different cytokines and chemokines (CCL3 and CCL4) that regulate immune response (35). In response to the stimulation by yeast cells, BMMCs showed increased production of IL-6 and IL-β that could not be induced by hyphae (35). *C. albicans* hyphae hide their β-glucan residues beneath a cover of mannoproteins on the cell walls, thus escaping Dectin-1-mediated phagocytosis and stimulating different cytokines production (36). Dectin-1, but not TLR2, is involved in the release of TNF-α through NF-кB/Syk pathway (35). The production of ROS and NO enhanced the ability to defense against *C. albicans* by MCs. When challenged with *C. albicans*, deletion of TLR2 or Dectin-1 significantly reduced NO levels, in comparison with wild type BMMCs. Whether Dectin-1 participates in the production of ROS remains controversial (16, 36).

Human MC immune responses towards *C. albicans* can be mainly divided into three phases. Human mast cell line HMC-1 degranulate in an early and direct response to fungal encounter, thus reducing the viability of *C. albicans* by 30% (37). Next, infected cell line HMC-1 release pro-inflammatory cytokines (e.g. IL-8) to recruit neutrophils, followed by the release of anti-inflammatory mediators, such as IL-16 and IL-1ra (37). MCs extracellular traps will be formed to contribute to fungal physical restriction, but not killing activity, as there was no significant difference in fungal viability in the presence or absence of nuclease (37). *C. albicans* can cause MCs death by different mechanisms, including the release of MC extracellular traps and rupture by the growth of internalized *C. albicans* hyphae, leading to a temporary immune response (37).

Although secretion of monocyte chemotactic protein 1 was not observed in infected MCs (37), they can produce short-lived soluble mediators to improve the crawling of tissue-resident macrophages and induce migration to *C. albicans* infection (38). Uninfected MCs were found to restrict macrophage phagocytosis of *C. albicans*, which is the result of maintaining a balance between the host and the fungus (38).

MCs exhibited dual effects in response to *C. albicans*, which is dependent on their protease content and site of *C. albicans* colonization. In mice, two types of MCs have been described as CTMCs, which originate from fetal liver progenitors and are mainly located in stromal tissue, and mucosal MCs (MMCs), which originate in the bone marrow and reside in the intestine and lung (39). Two similar types of MCs are also observed in humans (40). Different MCs types regulated by cytokine production (e.g. TGF-β, IL-9, IL-10) can discriminate between the fungal morphotypes, thereby promoting local inflammation or protective tolerance. Some studies have found that MMCs contribute to barrier function loss in leaky gut models (39) or higher sensitization against food antigen (41) in response to yeast stimulation. Whereas CTMCs induce local protective tolerance to infection by release of anti-inflammatory cytokine in response to hyphae (39). MCs promoting either inflammatory dysbiosis or tolerance were also observed in vulvovaginal candidiasis (42).

### MCs and *A. fumigatus* Infection

*A. fumigatus* is the most frequent opportunistic pathogenic species of the genus *Aspergillus* by far, responsible for about 90% of the cases of *Aspergillus* diseases (43). *A. fumigatus* is abundant in the environment, releasing spores (conidia) which are inhaled. After inhalation, patients with lung function or immune defenses impaired are unable to eliminate conidia which will germinate into hyphae that colonize the airways. *A. fumigatus* hyphae will penetrate pulmonary tissues causing invasive aspergillosis, the most severe disease caused by *Aspergillus* spp (44). *A. fumigatus* is known to contain 23 antigenic components that can induce the production of specific IgE (45). Classically, antigen-specific IgE which cross links FcϵRI can trigger MCs degranulation and increase IL-5 release (46). IL-5 is one of the important cytokines that can promote eosinophils migration to inflammation site, which are associated with severe asthma and allergic bronchial pulmonary aspergillosis (47, 48).With further studied, investigators found that mature *A. fumigatus* hyphae can directly stimulate MCs to degranulate in the absence of IgE (49). When cultivated with heat-killed *A. fumigatus* spores, HMC-1 cell line was activated to degranulation and release IL-5 (50). Taken together, the activation of MCs induced by *A. fumigatus* may be the initiator of the whole sensitization process. The studies concerning the signaling pathway of immune response of MCs in *A. fumigatus* infection are scarce. In dendritic cells, TLR2/MyD88 signaling pathway has been proved to regulate immune response to *A. fumigatus* conidia, which results in a development of Th2 response (51).

### MCs and *S. schenckii* Infection

*S. schenckii* is a common biphasic fungal pathogen. The incidence of sporotrichosis is increasing and has become an urgent global health problem (52). The World Health Organization classifies sporotrichosis as one of the Neglected Tropical Diseases which place a significant burden on individuals, families and society (53).

Studies have shown that MCs play a non-protective role in response to *S. schenckii* infection. In our previous study, we observed that MC-deficient C57BL/6-Kit^W^/Kit^W/v^ and MC-deficient Cpa3-Cre/Mcl-1^fl/fl^ (Hello Kitty, HK) mice had more serious inflammation symptoms with elevation of TNF-α and IL-6 in comparison with wild type mice (54). Rat peritoneal MCs can be activated by *S. schenckii* yeasts and release IL-6 and TNF-α *via* ERK pathway without degranulation (55, 56).

## Discussion

MCs are recognized as important effector in response to fungal infection. Their impacts can be protective and non-protective, depending on the pathogen and invasion site. Fungal recognition receptors commonly studied on MCs are TLR2/4 and Dectin-1.Upon activated by TLR/MyD88 or Syk pathway, MCs can produce specific cytokines and chemokines which can elicit a direct temporary immune response and recruit some neutrophils to the infection sites. MCs activation can enhance the immune response against infection. In contrast, the involvement of MCs in some immune responses may lead to more severe symptoms, such as intestinal barrier function loss, development of allergic bronchial pulmonary aspergillosis and increased area of inflammatory in S. schenckii infection, which suggests that MCs and their singling pathways are potential treatment regimens to prevent the clinically unwanted consequences. However, up to now, we still have a relatively poor understanding of the mechanism of MCs in response to common fungal infection. Many of the key signaling pathways involved in mediating selective cytokine and chemokine responses from MCs remain unknown. In the future, more *in vitro* and *in vivo* studies are urgently needed to further explore the role of MCs in response to fungal infection.

## Author Contributions

MY: substantial contributions to draft the manuscript. X-tS: drafted the article. BL: drafted the article. T-tL: participated in material collection. S-lL: participated in material collection. Z-tZ: substantial contributions to conception and reviewed the article critically for important intellectual content. All authors contributed to the article and approved the submitted version.

## Conflict of Interest

The authors declare that the research was conducted in the absence of any commercial or financial relationships that could be construed as a potential conflict of interest.
